# Could Galectin 3 Be a Good Prognostic Factor in Endometrial Cancer?

**DOI:** 10.3390/diagnostics10090635

**Published:** 2020-08-26

**Authors:** Aneta Cymbaluk-Płoska, Paula Gargulińska, Sebastian Kwiatkowski, Ewa Pius-Sadowska, Bogusław Machaliński

**Affiliations:** 1 Department of Gynecological Surgery and Gynecological Oncology of Adults and Adolescents, Pomeranian Medical University, Al. Powstańców Wielkopolskich 72, 70-111 Szczecin, Poland; p.gargulinska@wp.pl; 2 Department of Obstetrics and Gynecology, Pomeranian Medical University, Al. Powstańców Wielkopolskich 72, 70-111 Szczecin, Poland; kwiatkowskiseba@gmail.com; 3 General Pathology Department, Pomeranian Medical University, Al. Powstańców Wielkopolskich 72, 70-111 Szczecin, Poland; ewapius@wp.pl (E.P.-S.); machalin@pum.edu.pl (B.M.)

**Keywords:** galectin 3, endometrial cancer, prognostic factor, DFS, OS

## Abstract

Galectin 3 is a modulator of several basic biological functions. It may be involved in the development of obesity and type 2 diabetes—risk factors of endometrial cancer. The study involved 144 patients, after abrasion due to postmenopausal bleeding. Galectin 3 concentrations were quantified in serum by multiplex fluorescent bead-based immunoassays. Median serum galectin 3 concentrations revealed significant differences between FIGO III and IV vs. FIGO I and II patients. Statistically higher concentrations were reported for patients with lymph node metastases compared to patients without it (*p* = 0.001) as well as in patients with lymphovascular space invasion compared to patients without LVSI (*p* = 0.02). No statistically significant differences were observed for median of galectin 3 levels depending on the surgical procedure (laparoscopy vs. laparotomy, *p* = 0.0608). Patients with galectin 3 levels exceeding the median value were characterized by overall survival being shorter by 11.9 months. High levels of galectin 3 were correlated with shorter disease-free survival, the difference is up to 14.8 months. Galectin 3 can be an independent prognostic factor in patients with endometrial cancer. Among the recognized prognostic factors and the concentrations of the galectin 3 marker at the adopted time points, the univariate analysis showed a significant effect of staging, grading, and cutoff galectin 3 on the OS. For multivariate analysis, the galectin 3 cutoff point had the greatest significant impact on OS.

## 1. Introduction

Endometrial cancer is the most common cancer in perimenopausal women. Its prevalence is increasing in highly developed countries. The predominant type of endometrial cancer is endometrioid endometrial adenocarcinoma associated with obesity, diabetes, and metabolic syndrome disorders.

Although prognostic factors such as histopathological grade, clinical stage, histological type and presence of E and P receptors are well known in endometrial cancer, the search for novel proteins or glycoproteins to be used as better prognostic biomarkers continues. Hopes are raised in this context by galectin [1,2].

A total of 15 galectin isoforms are known, with galectin 3 being the most common protein of the lectin family in mammals. The molecular weight of Gal-3 is 35 kDa. The protein is coded by a single gene LGALS2 localized at position q21-q22 in chromosome 14 [3].

Galectin 3 is an important modulator of several basic biological functions. According to the most recent reports, galectin 3 may be involved in the development of obesity and type 2 diabetes. Levels of galectin 3 have been shown to be higher in obese and diabetic patients as well as in patients with carbohydrate metabolism disorders. Due to its domain-based structure consisting of the N-terminal domain encompassing the phosphorylation site and the C-terminal domain encompassing the conserved carbohydrate-recognition binding (CRB) domain, Gal-3 is capable of binding advanced end-products of glycation and lipoxidation, which are accumulated in target organs to exert toxic effects by activating pro-inflammatory and pro-oxidative pathways [4]. This is related to numerous pathologies, particularly in the course of long-term complications of diabetes or metabolic syndrome. In addition to the known inflammatory effects of galectin 3, also described was its proliferation-stimulating involvement in connective tissue fibrosis as well as in angiogenesis and apoptosis [5,6,7]. Due to the enormous role of obesity and diabetes as risk factors for endometrial cancer and the fact that galectin 3 is involved in carcinogenesis, we decided to examine its potential as a promising prognostic factor for endometrial cancer.

## 2. Material and Methods

The study involved 144 patients, who were undergoing abrasion due to postmenopausal bleeding.

All the patients that were involved in the research signed informed consent and agreed to all the requirements. The study protocol was approved by the Ethical Committee of the Pomeranian Medical University.

Height and body weight were measured in patients who were eligible for the study. On the basis of measurement results, BMI values were calculated for each patient.

Following formula was used to calculate BMI:

$$\mathrm{BMI}\;=\;\frac{\mathrm{weight}\;\left[\mathrm{kg}\right]}{\mathrm{height}\;{\left[m\right]}^{2}}$$

Patients were assigned to 2 groups depending on their BMI value:

BMI < 30, n = 77

BMI > 30, n = 67

Patients were asked about their medical history, chronic diseases, and medications they were taking. Due to the presence of type 2 diabetes, patients were divided into two groups:

DM type 2—yes, n = 71

DM type 2—no, n = 73

Patients were also divided into two groups on the basis of their hypertension history:

HA—yes, n = 89

HA—no, n = 55

Due to the indicators of inflammatory parameters, patients were divided into two groups:

CRP < 5, n = 65

CRP > 5, n = 79

In the study, patients were divided into two groups depending on the use of hormonal replacement therapy

HRT—no, n = 86

HRT—yes, n = 58 (32 patients with the transdermal route and 26 patients with the oral route)

Among patients taking HRT:

- 55 had two-component HRT—estrogen/progesterone
- 3 patients were taking tibolone—estrogen/progesterone/androgen

Moreover, each patient was asked about habits. There were a few patients who consumed alcohol occasionally. There was no division of patients due to alcohol consumption. Group was divided depending on the smoking status.

Smokers—yes, n = 63

Smokers—no, n = 81(1)

Detailed patient characteristics relating to the endometrial cancer risk factors are shown in Table 1.

Patients qualified for the study underwent the following procedures: abrasion, hysteroscopy, or radical surgery. Radical operations mean total hysterectomy and bilateral salpingo oophorectomy with lymph nodes concerned only the study group, i.e., patients with histopathologically confirmed diagnosis of endometrial cancer after abrasion or hysteroscopy. The control group included patients after abrasions or hysteroscopies with histopathologically normal endometrium.

Two groups of patients were established after histopathological results were obtained. Study group A and control group B.

Group A—Endometrial cancer patient, n = 82.

Group B—Patients with normal endometrium, n = 62.

Uni- and multivariate Cox regression analyses were carried out to assess the widely recognized standard prognostic factors and the galectin 3 levels in terms of their impact on DFS and OS values. The study group included 64 patients with endometrial endometrioid carcinoma and 18 patients with nonendometrial endometrioid carcinoma—Table 2.

Excluded from the study were patients with a history of treatment for other oncology disease or incomplete data.

During routine preoperative examinations, 5 mL of blood were collected from each patient who agreed for the examination. After centrifuging, obtained serum was stored in a freezer at −70 °C.

To measure galectin 3 level, multiplex fluorescent bead-based immunoassays (Luminex Corporation, Austin, TX, USA) and commercial Bio-Plex Pro RBM Human Metabolic Panel 2 (Bio-Rad, Hercules, CA, USA) were used. Precisely, 30 μL of standard, control, and action were added on the plate. Next, 10 µL blocker with 10 µL downstream of the antibody capture multiplex were added to all wells on the plate. Samples were incubated for 1 h during shaking at room temperature. After this stage, the wells were washed three times using the test buffer. Next, using pipette, 40 μL of antibody detection cocktail was added to each well. The plate was incubated for 1 h at room temperature. After the shaking stage, a streptavidin-phycoerythrin mixture was added. Next, samples were again incubated at the room temperature, in the dark, for 30 min. Another stage was to wash the microspheres. Buffer was added again and shaken for 30 s at room temperature. The plate was analyzed on a Luminex analyzer.

For statistical calculations, the Statistica 10 PL software was used. For the basic descriptive analysis characterizing the examined group of patients, min, max, range, mean, and median values were used. The distribution of data in the studied group of patients was not normal and homogeneous, therefore nonparametric tests were used. For the comparison between the two groups, Mann–Whitney’s U test was used.

Uni- and multivariate analyses were performed using the Cox regression model. Parameters included in multivariate Cox analysis included age, FIGO stage, tumor grade, as well as median, cutoff, 75th percentile, and 95th percentile of galectin level.

## 3. Results

### 3.1. Characteristics of Patients in Terms of Endometrial Cancer Risk Factors

The age, hormonal status, and endometrial cancer risk factor characteristics of all study patients are presented in Table 1.

Statistically significant correlations were found between BMI and galectin 3 concentration, r = 0.809 for *p* = 0.004.

Statistically more significant galectin 3 serum levels were observed in nulliparous vs. multiparous women (*p* = 0.043), obese vs. normal body weight patients (*p* = 0.021), type 2 diabetes patients vs. nontype 2 diabetes patients (*p* = 0.018), as well as in patients with elevated C-reactive protein (CRP) levels (*p* = 0.036). No statistically significant differences were observed between patients depending on their hormonal status, hormone replacement therapy status, or smoking status. In addition, the median serum concentration of galectin 3 was significantly higher in the group of patients with endometrial cancer as compared to patients with normal endometrium (*p* = 0.002).

### 3.2. Comparative Analysis of Galectin 3 Concentrations Depending on the Stage and the Grade of Cancer

Table 3 presents differences in the medians of serum galectin 3 concentrations in patients with endometrioid vs. nonendometrioid cancers of endometrium. Detailed analysis of median serum galectin 3 concentrations depending on endometrial cancer stage revealed significant differences between FIGO III and IV vs. FIGO I and II patients. In addition, statistically higher concentrations were reported for patients with lymph node metastases as compared to patients without lymph node metastases (*p* = 0.001) as well as in patients with lymphovascular space invasion (LVSI) as compared to patients without LVSI (*p* = 0.02). A significantly higher median of galectin 3 levels was also observed in patients with low-differentiated G3 cancer as compared to the medial of galectin 3 levels in patients with well-differentiated cancer. Results of serum galectin 3 concentrations in patients with particular advancement are presented in Table 4. However, no statistically significant differences were observed for median of galectin 3 levels depending on the surgical procedure (laparoscopy vs. laparotomy, *p* = 0.0608).

### 3.3. The Analysis to Confirm the Applicability of Galectin 3 as a Good Diagnostic Test

**Figure 1:**
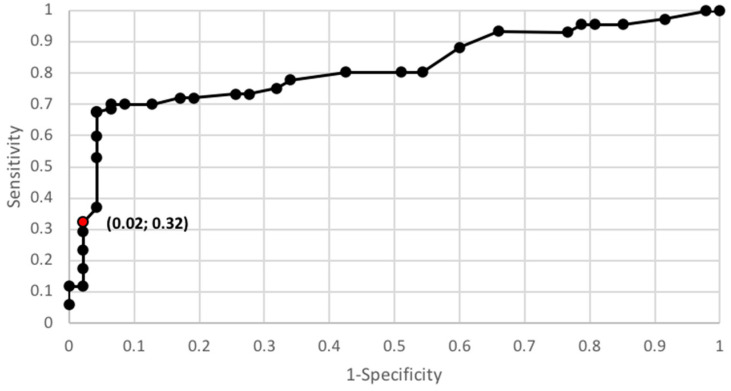
Galectin 3 protein ROC curve depending on grading (AUC = 0.9).

**Figure 2:**
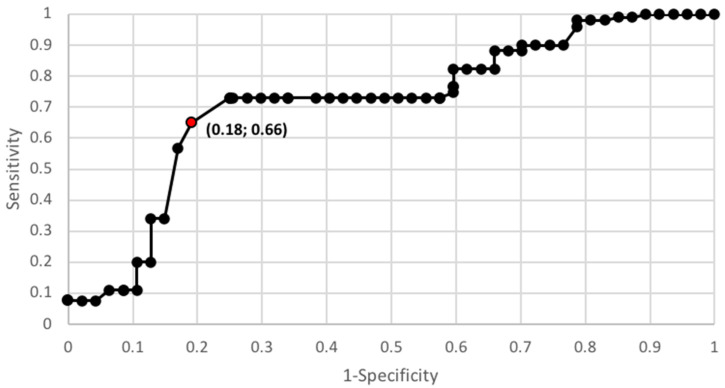
Galectin 3 protein ROC curve depending on staging (AUC = 0.89).

In order to verify the usefulness of galectin 3 as an early marker for endometrial cancer, the ROC curve was used, and the AUC was calculated for this curve. The AUC for galectin 3 was 0.68 in the total population of patients, including 0.61 in premenopausal patients and 0.74 in postmenopausal patients. The AUC value was 0.89 when taking into account the patient population being divided into two clinical staging groups of FIGO III and IV vs. FIGO I and II and 0.90 when patient population was divided according to histopathological grade G1 vs. G2 and G3—Figure 1 and Figure 2.

When estimating the sensitivity and specificity of galectin 3 regardless of patient hormonal status, the sensitivity was found to be higher than the specificity (0.97 vs. 0.65, respectively).

### 3.4. DFS and OS

The study cohort of patients was dichotomized according to different galectin 3 level cutoff values, including:

- median baseline galectin 3 level
- 75th percentile of baseline galectin 3 levels
- 95th percentile of baseline galectin 3 levels
- baseline galectin 3 level cutoff level.

**Figure 3:**
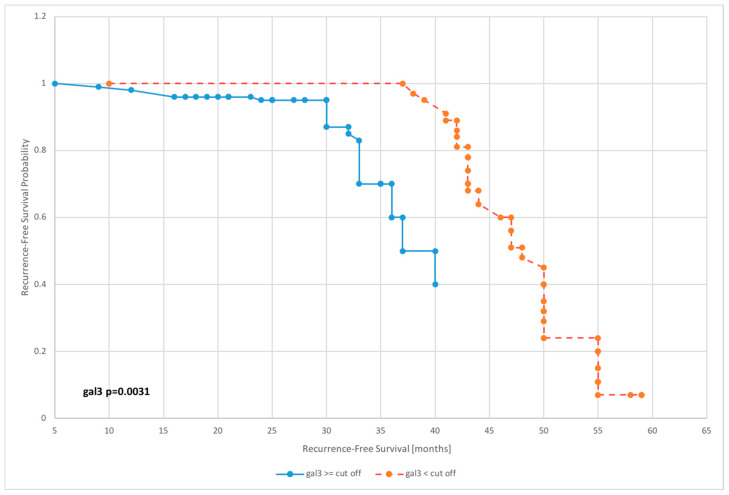
Kaplan–Meier recurrence-free survival curves for endometrial cancer patients based on serum galectin 3 level for cutoff.

**Figure 4:**
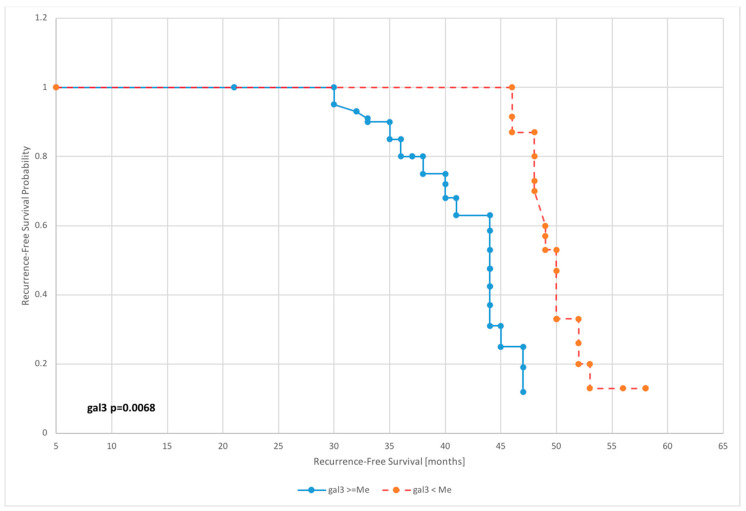
Kaplan–Meier recurrence-free survival curves for endometrial cancer patients based on serum galectin 3 level for median.

**Figure 5:**
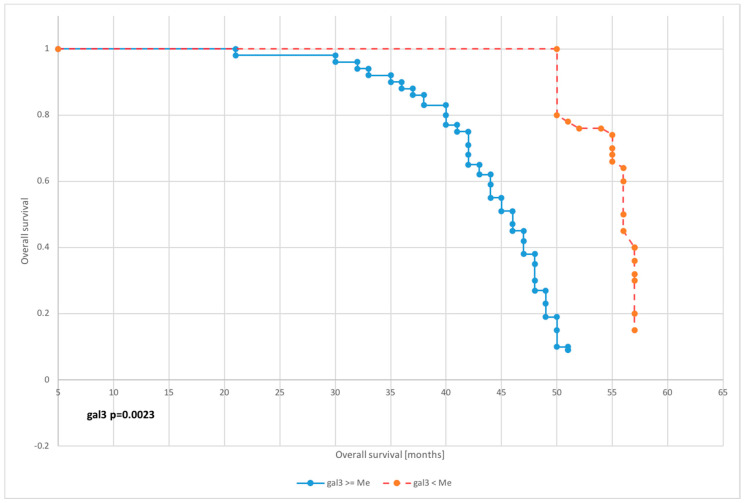
Kaplan–Meier overall-free survival curves for endometrial cancer patients based on serum galectin 3 level for cutoff.

**Figure 6:**
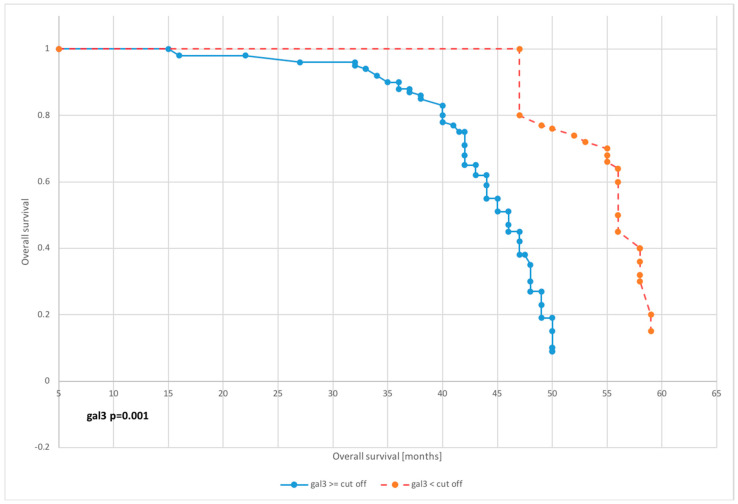
Kaplan–Meier overall-free survival curves for endometrial cancer patients based on serum galectin 3 level for median.

The survival analysis was carried out on the basis of Kaplan–Meier curves with the most interesting results of proportional hazards regression shown in Figure 3, Figure 4, Figure 5 and Figure 6.

Galectin 3 levels affected overall survival of patients in nearly all dichotomized subgroups excluding subgroups dichotomized according to the 75th percentile value (*p* = 0.1542).

The mean survival for patients with preoperative galectin 3 levels below the median was 57.1 months, while for those with level above median was 50.3 months. Furthermore, for patients with galectin 3 serum concentration below cutoff point, mean survival was 58.4, whereas for those with level above cutoff was 48.2—Table 3.

### 3.5. Univariate and Multivariate Logistic Regression Analysis

Uni- and multivariate Cox regression analyses were carried out to assess the widely recognized standard prognostic factors and the galectin 3 levels in terms of their impact on DFS and OS values.

Both the uni- and the multivariate analyses revealed that for all the prognostic factors in use, clinical stage and histopathological grade had a significant impact on DFS. However, we found that the factors with the greatest impact on overall survival were age and cancer stage. Univariate analysis revealed that galectin 3 had a significant impact on selected survival parameters in relation to DFS (output values above and below the median: HR = 1.41, *p* = 0.003; output values above and below the 75th percentile: HR = 1.62, *p* = 0.0042; output values above and below the 95th percentile: HR = 1.71, *p* = 0.0031; and values below and above the cutoff level: HR = 1.54, *p* = 0.0004) and OS (output values above and below the median: HR = 1.22, *p* = 0.0267; output values above and below the 95th percentile: HR = 1.30, *p* = 0.0076; and values below and above the cutoff level: HR = 1.48, *p* = 0.0091). A comparative assessment of the impact on DFS and OS in uni- and multivariate analyses is presented in Table 5. No statistically significant differences were found in the period to relapse and in overall survival depending on the type of surgical procedure (laparoscopy vs. laparotomy)—Table 6.

## 4. Discussion

In 2012, a rapid increase was observed in the incidence of endometrial cancer. The problem concerned Central America, South America, as well as Central and Eastern Europe. This can be linked to the epidemic of obesity and related metabolic disorders known to be risk factors for endometrial cancer.

Galectin 3 has now been identified as a modulator for both metabolic and inflammatory processes [8,9,10,11].

Like leptin, galectin 3 is strictly associated with the development of metabolic disorders. It plays a key role in the pathogenesis of insulin resistance, type 2 diabetes, and lipid disorders. Galectin 3 is active within the adipose tissue [9].

What is known about galectin 3 is that it is responsible for initiation of inflammatory macrophage infiltration within the adipose tissue; this is in turn correlated with both carbohydrate and lipid metabolism disorders. Agents responsible for these disorders include cytokines and fatty acids being released as the effect of galectin 3, leading to insulin resistance and cellular dysfunction. Obesity is associated with chronic inflammation initiated by certain cytokines interacting with the RAGE receptor [12].

In our studies, significantly higher levels of galectin 3 were found in patients with obesity and type 2 diabetes. Galectin 3 levels were higher in patients with elevated inflammatory markers (statistical significance could not be demonstrated). McCullough et al., Yu et al., and Bertocchi demonstrated a positive correlation between galectin 3 levels and C-reactive protein (CRP) in autoimmune diseases such as rheumatoid arthritis [8,13,14]. In addition, according to Gao et al. and Clementy et al., levels of galectin 3 are higher in patients with asthma as well as patients with confirmed atherosclerosis [15,16].

Galectin 3 also appears to play a significant role in proliferation, angiogenesis, and metastasis of tumors [17,18,19,20].

Our research failed to confirm that galectin 3 can be used as a good diagnostic test for use in endometrial cancer screening. AUC was at the level of 0.64. In addition, in the face of reports by Iurisci et al. who found that serum galectin 3 levels were higher in 7 studied tumor types, namely, breast cancer, stomach cancer, lung cancer, ovarian cancer, melanoma, Hodgkin’s lymphoma, as well as non-Hodgkin’s lymphoma compared to the control group; galectin 3 cannot be used as a diagnostic biomarker [21].

One of the well-known galectins is galectin 7, which is tissue specific. The role of galectin 7 may be pro- or anticarcinogenesis. It has been proven to be procarcinogenic in breast, ovarian, and clear cell kidney cancer, while its anticancer effect has been demonstrated in gastric, prostate, and colon cancer [22]. Menkhorst et al. demonstrated on endometrial cancer cell lines that galectin 7 influences cell proliferation and migration. It does not affect apoptosis. Cell migration and proliferation was significantly greater with the G3 grading than with the G1 grading. The conclusions drawn by the authors are that galectin 7 have possible influence on the formation of distant metastases in endometrial cancer [23].

Galectin 3 levels were significantly higher for tumors of higher stage as well as for cancers which had spread into the lymph nodes, possibly indicating that galectin 3 plays a role in the spread of cancer.

Completely different results suggestive of the great proapoptotic effect of galectin 3 can be found in the study by AL Maghrabi [24]. As reported by the author, galectin 3 levels were strictly associated with tumor suppression. Lower immunohistochemical expression was observed for endometrial tumors characterized by low differentiation and higher clinical stage. This is not consistent with the results obtained by Lambropoulou et al. where high expression of galectin 3 was independently associated with the infiltration depth and the histological grade of the tumor [25]. Both proliferative and apoptotic effects of galectin 3 are well illustrated by Hafiz et al. who demonstrated that galectin 3 as expressed on the surface of cells is involved in homotypic cell adhesion via binding to soluble complementary glycoconjugates [26,27]. Interactions between metastatic neoplastic cells with vascular endothelium are critical in the early stages of tumor metastasis [28,29]. Galectin 3 mediates homotypic and heterotypic aggregation of cells and promotes interactions between neoplastic and endothelial cells, leading to angiogenesis and metastasis [5].

With regard to its impact on apoptosis, galectin 3 is known to generally remain in equilibrium between intracellular antiapoptotic activity and proapoptotic extracellular activity.

This equilibrium is maintained via two main signaling pathways, one of them involving the FAS (apo-1/CD95) death receptor and the other involving TNF-related apoptosis-inducing ligand (TRAIL or Apo2-L), known for its external apoptotic signals. Matarrese et al. observed that overexpression of galectin 3 led to increased adhesion and inhibited apoptosis of breast cancer cells [30].

Multivariate analysis carried out as a part of our study revealed that galectin 3 may be an independent prognostic factor for endometrial cancer.

Particularly, large impact on overall survival was noted for the galectin 3 median concentration, the 95th percentile of the initial galectin 3 concentration and the galectin 3 cutoff point. In the case of disease-free time, only the concentration above the median and the cutoff point for galectin 3 had a significant effect. In the univariate analysis, statistical significance affecting DFS and OS was found for all so far recognized prognostic factors for endometrial cancer and the galectin levels 3. Only the 75th percentile of the baseline galectin 3 was not statistically significant for the overall patients’ survival.

No data are available from any studies for any comparison of serum galectin 3 levels in this type of cancer.

Studies in urinary bladder cancer as conducted by Gendy et al. suggest that galectin 3 might be a promising new biomarker [31]. In addition, it was reported that galectin 3 can be used as a prognostic factor rather than a diagnostic marker in hepatocellular carcinoma. The role of galectin in pancreatic, breast, colon, and thyroid cancer is unclear; in contrast, studies on gastric cancer revealed that it is impossible to use galectin 3 for clinical diagnostics as well as for prediction of disease recurrence and survival [32,33].

In our studies, higher serum galectin 3 levels were observed in patients with lymph node metastases. The most likely explanation appears to be related to overexpression of mucin 1 on the surface of tumor cells. Galectin 3 is a natural ligand for mucin 1; after binding, it triggers the endothelial growth factor and epidermal pathways opening up the possibilities for cancer spread. This is not the only explanation of the role of galectin 3 in tumor metastasis. Two experimental works were published that show the role of galectin 3 in the in vivo spread of the tumor [5]. Metastasis is triggered by galectin 3 interacting with extracellular matrix proteins such as laminin, fibronectin, and vitronectin [34,35].

In addition, galectin 3 is capable of mediating cell-to-cell adhesion by interacting with complementary glycoproteins [33] indicating that galectin 3 is involved in the development of neoplastic emboli and the spread of neoplastic cells in circulation. In our studies, galectin 3 levels were strictly correlated with disease-free survival and overall survival of patients. No similar studies assessing the serum galectin 3 levels in endometrial cancer are available in the literature.

In a study by Brustmann et al., galectin 3 expression during immunohistochemical staining was much greater in pathological hyperplasia and endometrial carcinoma than in the control group [36]. Completely different reports are presented by Ege et al. and Van den Brule et al., who found a significantly lower expression of galectin 3 in endometrial carcinomas than in the control group [37,38]. These contradictory results may be due to the fact that tumor cells are subject to the surrounding microenvironment when they metastasize and this may have a significant impact on the spread of cancer. Nevertheless, due to our research, we believe that an increased level of galectin 3 serum affects the number and speed of metastasis. In order to deepen our research, we plan to check the serum level of galectin 3 in the next study, assess its expression in the primary tumor, and assess the number of galectin receptors in the lymph nodes and the top of vagina to check the actual pathway of tumor metastasis, depending on galectin 3.

## 5. Conclusions

Galectin 3 can be an independent prognostic factor in patients with endometrial cancer, which would allow individualization of treatment in patients with this type of cancer.

## Figures and Tables

**Table 1:** Serum galectin 3 levels in patients with risk factors for endometrial cancer.

	Characteristic	Median	95% CI
144	Age	56.2	(48.2–61.3)
36	Nullipara	17.1	(14.0–26.2)
81	Never smoking	16.3	(15.1–23.8)
63	Current smoker	17.2	(14.9–19.6)
71	Diabetic (yes)	21.1	(16.3–22.8)
73	Diabetic (no)	16.8	(14.2–17.1)
86	Hormonal replacement therapy (never)	17.2	(15.5–19.9)
58	Hormonal replacement therapy	18.4	(16.7–21.8)
89	Hypertension (yes)	19.1	(16.3–23.0)
55	Hypertension (no)	17.2	(15.5–20.7)
67	Obesity (yes)	21.6	(18.0–24.6)
77	Obesity (no)	16.4	(16.2–18.1)
65	CRP < 5	14.9	(13.2–19.1)
79	CRP > 5	18.6	(16.3–22.5)

**Table 2:** Patients with endometrial cancer divided into subgroups.

Subgroups	Distribution	Numbers
The histopathological type	Endometrial endometrioid adenocarcinoma	64
	Nonendometrioid adenocarcinoma	18
Histopathological grade of the tumor	G1	26
	G2	35
	G3	21
Clinical stage of the tumor	FIGO I and II	61
	FIGO III and IV	21
Lymphovascular space (LVSI) invasion	Yes	45
	No	39
Lymph node metastases	With lymph node metastases	33
	Without lymph node metastases	51

**Table 3:** Survival time for patients depending on the median and cutoff level of galectin 3.

	Median <17.8	Median >=17.8	Cutoff <16.2	Cutoff >=16.2
36 months	n = 52	n = 14	n = 55	n = 11
5 years	n = 45	n = 8	n = 46	n = 7
Mean survival	57.1 months	50.3 months	58.4 months	48.2 months

**Table 4:** Relationship between galectin 3 serum concentration and pathological stage and findings.

n Patients	Pathological Stage	Pathological Findings	Median (95%CI) [ng/mL]
7	IA	LVSI (−), G1	15.7 (13.1–16.0)
5	IA	LVSI (+), G1	15.6 (14.4–19.2)
9	IA	LVSI (+), G2-G3	16.8 (16.5–18.9)
3	IB	LVSI (−), G1	17.2 (16.1–19.5)
2	IB	LVSI (+), G1	16.5 (15.0–17.1)
8	IB	LVSI (−), G2-G3	17.9 (17.8–19.2)
9	IB	LVSI (+), G2-G3	18.4 (17.2–20.3)
2	IIA	LVSI (−), G1	18.1 (17.2–20.6)
2	IIA	LVSI (−), G2-G3	19.4 (15.8–21.0)
3	IIA	LVSI (+), G1	18.6 (17.1–20.9)
3	IIA	LVSI (+), G2-G3	20.2 (19.4–23.1)
2	IIB	LVSI (−), G1	19.9 (18.7–21.6)
2	IIB	LVSI (+), G1	20.7 (19.5–22.1)
2	IIB	LVSI (−), G2-G3	21.5 (19.8–22.3)
12	IIIA	LVSI (+), G2-G3	22.2 (19.7–23.1)
6	IIIB	LVSI (+), G2-G3	22.6 (18.9–22.8)
3	IVA	LVSI (+), G2-G3	25.2 (21.6–24.8)

**Table 5:** Univariate and multivariate statistical analysis.

	**Univariate Analysis (Cox Regression Model)**					
	**PFS**			**OS**		
	**HR**	**95% CI**	***p***	**HR**	**95% CI**	***p***
**Age**	2.11	(1.6–2.4)	0.003	1.38	(1.20–1.53)	0.0367
**Stage III, IV vs. I, II**	3.63	(3.0–3.86)	0.002	3.04	(2.78–3.21)	0.0113
**Grade 3 vs. 1**	2.09	(1.71–2.61)	0.021	1.94	(1.69–2.09)	0.0062
**Galectin 75th percentile**	1.62	(1.38–1.68)	0.0042	1.38	(1.16–1.41)	0.0562
**Galectin 95th percentile**	1.71	(1.55–1.82)	0.0031	1.30	(1.22–1.49)	0.0076
**Galectin median**	1.41	(1.31–1.66)	0.003	1.22	(1.06–1.37)	0.026
**Galectin cutoff**	1.54	(1.43–1.61)	0.0004	1.48	(1.24–1.62)	0.0091
	**Multivariate Analysis (Cox Regression Model)**					
	**DFS**			**OS**		
	**HR**	**95% CI**	***p***	**HR**	**95% CI**	***p***
**Galectin median**	1.18	(0.96–1.32)	0.026	1.21	(1.08–1.3)	0.0216
**Galectin 75th percentile**	1.23	(1.12–1.39)	0.612	1.12	(1.04–1.34)	0.1542
**Galectin 95th percentile**	1.08	(0.99–1.21)	0.054	1.29	(1.12–1.46)	0.048
**Galectin cutoff**	1.63	(1.41–1.80)	0.008	1.46	(1.20–1.55)	0.022

DFS—disease-free survival; OS—overall survival; HR—hazard ratio.

**Table 6:** Comparison between different subgroups of patients according to the type of surgery.

Laparoscopy			Laparotomy			*p*
G1 n = 20	G2 n = 22	G3 n = 5	G1 n = 6	G2 n = 13	G3 n = 16	–
FIGO 1 + 2 n = 41	FIGO 3 + 4 n = 6		FIGO 1 + 2 n = 20	FIGO 3 + 4 n = 15		–
Lymph nodes invasion YES n = 11	Lymph nodes invasion NO n = 36		Lymph nodes invasion YES n = 22	Lymph nodes invasion NO n = 15		–
DFS n = 27.6			DFS n = 28.1			0.0612
OS n = 49.3			OS n = 52.6			0.0508
